# Association of the Red Blood Cell Distribution Width-to-Albumin Ratio with Renal Outcomes in Chinese Patients with IgA Nephropathy

**DOI:** 10.3390/jcm15208106

**Published:** 2026-10-19

**Authors:** Lingqiu Dong, Zhanxiong Shen, Siqing Wang, Yi Tang, Wei Qin

**Affiliations:** Division of Nephrology, Department of Medicine, West China Hospital, Sichuan University, Chengdu 610041, China

**Keywords:** red blood cell distribution width-to-albumin ratio, RAR, IgA nephropathy, renal survival

## Abstract

**Background**: The red blood cell distribution width-to-albumin ratio (RAR) is a novel composite biomarker that integrates hematological, inflammatory, and nutritional information, but its association with renal outcomes in immunoglobulin A nephropathy (IgAN) remains unclear. This study aimed to evaluate the association between RAR and long-term renal outcomes in patients with biopsy-proven IgAN. **Methods**: This study was a retrospective analysis of an established observational cohort, including 1169 patients with biopsy-proven IgAN. RAR was analyzed both categorically, using a receiver operating characteristic (ROC)-derived cutoff, and continuously. Propensity score matching (PSM) was performed at a 1:1 ratio. Kaplan–Meier analysis and multivariable Cox proportional hazards models were used to evaluate the association between RAR and the composite renal endpoint. Restricted cubic spline (RCS) analysis was used to assess the shape of the association. **Results**: Before PSM, higher RAR was independently associated with the composite renal endpoint in the fully adjusted model (HR 1.880, 95% CI 1.222–2.893; *p* = 0.004). The association remained significant after PSM (HR 2.128, 95% CI 1.067–4.243; *p* = 0.032). When analyzed continuously, RAR was associated with a higher risk of the composite endpoint before PSM (HR 1.371, 95% CI 1.099–1.711; *p* = 0.005) and after PSM (HR 1.989, 95% CI 1.199–3.299; *p* = 0.008). RCS analyses showed significant overall associations before and after PSM, without significant evidence of nonlinearity. **Conclusions**: Higher RAR was independently associated with adverse renal outcomes in patients with IgAN. Further external validation and large multicenter prospective studies are needed to determine its clinical value.

## 1. Introduction

IgA nephropathy (IgAN) is the most common primary glomerular disease worldwide, especially in East Asia [1,2]. It is characterized by predominant mesangial IgA deposition, with heterogeneous clinical courses, ranging from persistent urinary abnormalities, including hematuria with or without proteinuria, to progressive loss of kidney function and kidney failure [3]. Approximately 20–40% of patients were reported to progress to end-stage renal disease (ESRD) within 10–20 years after diagnosis [4]. This wide variation in disease courses highlights the need for simple laboratory parameters that may help identify patients with a higher risk of renal progression.

The pathogenesis of IgAN is commonly explained by the “multi-hit” hypothesis, in which abnormal immune responses, inflammation, and subsequent renal injury all play important roles [5,6]. Recent studies have explored several indices calculated from routine laboratory measurements to assess inflammation, metabolic changes, and nutritional status in patients with IgAN [7,8,9,10]. These findings support the potential value of simple composite indices in renal risk assessment. However, most of these markers mainly represent one or two biological domains. A readily available index that simultaneously incorporates hematological changes, inflammation or oxidative stress, and nutritional status remains less well studied.

The red blood cell distribution width-to-albumin ratio (RAR) combines information from red blood cell distribution width (RDW) and serum albumin (ALB) in a single index. Higher RAR has been associated with adverse outcomes in the general population and in patients with cardiovascular disease, neurodegenerative disorders, diabetes-related complications, and chronic kidney disease (CKD) [11,12,13,14]. RDW, which reflects heterogeneity in erythrocyte size, has traditionally been used in the differential diagnosis of anemia. Beyond its hematological role, elevated RDW has also been associated with chronic inflammation, oxidative stress, impaired erythropoiesis, and reduced erythrocyte deformability [15]. Previous studies have further linked higher RDW levels with adverse outcomes in cardiovascular disease, gastrointestinal disorders, CKD, and dialysis populations [16,17,18,19,20,21]. Serum albumin is widely used as a marker of nutritional status and is also affected by systemic inflammation [22]. However, previous CKD studies mainly involved heterogeneous patient populations with different underlying causes of kidney disease and did not specifically evaluate biopsy-proven IgAN. Because the progression of IgAN is closely related not only to clinical factors such as proteinuria, kidney function, and blood pressure, but also to disease-specific pathological findings such as Oxford MEST-C lesions, the prognostic significance of RAR in IgAN cannot be directly inferred from general CKD populations. Whether RAR remains associated with renal outcomes after accounting for these established clinical and pathological factors remains unclear. Therefore, we investigated the association between RAR and renal outcomes in patients with biopsy-proven IgAN.

## 2. Materials and Methods

### 2.1. Study Population

**Figure 1:**
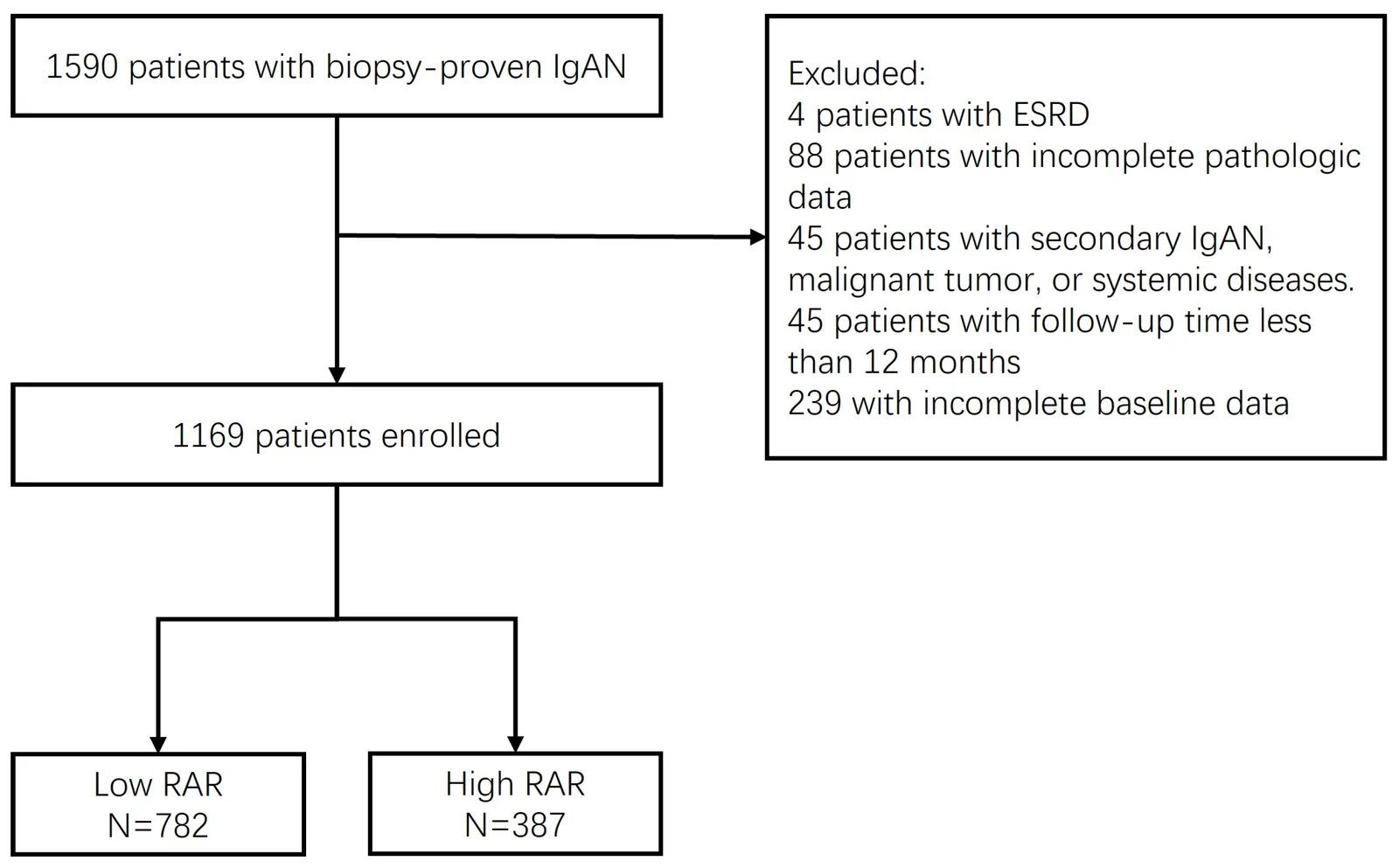
Flowchart of enrolled patients in this study. Abbreviations: IgAN, IgA nephropathy; ESRD, end-stage renal disease; RAR, red blood cell distribution width-to-albumin ratio.

This study was a retrospective analysis of an established observational cohort of patients with biopsy-proven IgAN at West China Hospital, Sichuan University. A total of 1590 patients were enrolled in the cohort between 2009 and 2018 [7]. The original study was performed according to the Helsinki Declaration and was authorized by the Ethics Committee of West China Hospital of Sichuan University (2019-33). Informed consent was obtained from all subjects involved in the study at the time of cohort enrollment [7]. The present analysis was conducted using previously collected data and was covered by the original informed consent and ethical approval. Therefore, no additional informed consent was required. The exclusion criteria were as follows: (1) secondary IgAN, including systemic lupus erythematosus, Henoch–Schonlein purpura, chronic liver disease and diabetes mellitus; (2) insufficient data; (3) biopsy specimens with fewer than eight glomeruli, (4) follow-up shorter than 12 months; and (5) estimated glomerular filtration rate (eGFR) < 15 mL/min/1.73 m^2^. Finally, 1169 patients were included (Figure 1).

### 2.2. Clinical Data Collection

Demographic and clinical data were obtained from the electronic medical record system at the time of renal biopsy, including age, sex, systolic blood pressure (SBP), diastolic blood pressure (DBP), hemoglobin (Hb), serum creatinine, eGFR, ALB, RDW, total triglycerides (TG), total cholesterol (TC), high-density lipoprotein cholesterol (HDL-C), low-density lipoprotein cholesterol (LDL-C), 24 h proteinuria, urinary red blood cell count (uRBC), and serum uric acid (UA). The eGFR was calculated using the Chronic Kidney Disease Epidemiology Collaboration (CKD-EPI) equation [23]. Chronic kidney disease stage was classified according to baseline eGFR according to the Kidney Disease: Improving Global Outcomes (KDIGO) guideline [24]. Anemia was defined as Hb < 120 g/L in men and <110 g/L in women [25]. Hypoalbuminemia was defined as ALB < 30 g/L [26,27]. Hematuria was defined as uRBC > 5/HPF [28,29]. Hypertriglyceridemia was defined as TG ≥ 2.3 mmol/L, and hypercholesterolemia as TC ≥ 6.2 mmol/L [30].

### 2.3. Pathological Data Collection

Renal biopsy specimens were evaluated according to the Oxford MEST-C classification [31]. Pathological lesions included mesangial hypercellularity (M), endocapillary hypercellularity (E), segmental glomerulosclerosis (S), tubular atrophy/interstitial fibrosis (T), and cellular or fibrocellular crescents (C).

### 2.4. Treatment and Endpoint

All patients received supportive therapy based on an angiotensin-converting enzyme inhibitor (ACEI) or angiotensin receptor blocker (ARB). Corticosteroids were administered as prednisone at 0.5–1 mg/kg/day with gradual tapering over 6–8 months. Additional immunosuppressive therapy, including cyclophosphamide (2 mg/kg/day for 3 months) or mycophenolate mofetil (1–2 g/day for 6–8 months), was prescribed according to clinical severity and pathological findings. The study endpoint was the composite endpoint, which indicates ESRD (eGFR less than 15 mL/min/1.73 m^2^ or maintenance renal replacement treatment) and/or an eGFR decrease of more than 50% compared with baseline.

### 2.5. RAR Calculation and Discriminatory Assessment

RAR was calculated from baseline laboratory measurements obtained at the time of renal biopsy: RDW (%)/ALB (g/L). Receiver operating characteristic (ROC) curve analysis was performed to evaluate the discriminatory ability of RAR for the composite renal endpoint. The area under the curve (AUC) was calculated, and the cutoff for RAR was determined by the maximum Youden index (sensitivity + specificity − 1) [32].

### 2.6. Statistical Analysis

Normally distributed continuous variables were presented as the mean ± standard deviation (SD) and were compared using Student’s *t* test. Non-normally distributed continuous data were presented as medians and interquartile ranges and compared using the Mann–Whitney U test. Categorical variables are presented as numbers and percentages and were compared using the χ^2^ test or Fisher’s exact test.

ROC curve analysis was used to evaluate the discriminatory ability of RAR for the composite renal endpoint. The optimal cutoff was identified using the maximum Youden index. AUCs for RAR, RDW, and ALB were compared using the DeLong test.

To reduce baseline differences between the low- and high-RAR groups, 1:1 propensity score matching (PSM) was conducted. The propensity score was estimated using multivariable logistic regression including baseline variables that differed between the low- and high-RAR groups. Patients were then matched without replacement with a caliper of 0.2 standard deviations of the logit of the propensity score. Matching quality was assessed using standardized mean difference (SMD), with values < 0.20 considered indicative of adequate covariate balance [33]. Follow-up duration was not included in the propensity score model because it was not a baseline characteristic.

Univariate and multivariable Cox proportional hazards models were used to evaluate associations with the composite renal endpoint. RAR was analyzed as both continuous variable and categorical variable (lower RAR vs. higher RAR) in Cox regression models. The proportional hazards assumption was assessed using scaled Schoenfeld residuals. For covariates showing evidence of non-proportionality, time-varying effects were incorporated by introducing interactions with log(time) [34,35]. Restricted cubic spline (RCS) analysis was performed to assess the shape of the association between RAR and the composite renal endpoint. Overall and nonlinearity *p* values were calculated. Renal survival between groups was compared via the Kaplan–Meier method and compared using the log-rank test. Subgroup analyses were performed according to sex, proteinuria, anemia, eGFR, and treatment, and potential differences across subgroups were assessed using interaction tests.

The analysis was performed using R version 4.5.2, and a two-tailed *p* < 0.05 was considered as statistically significant.

## 3. Results

### 3.1. Demographic and Clinicopathological Characteristics

**Figure 2:**
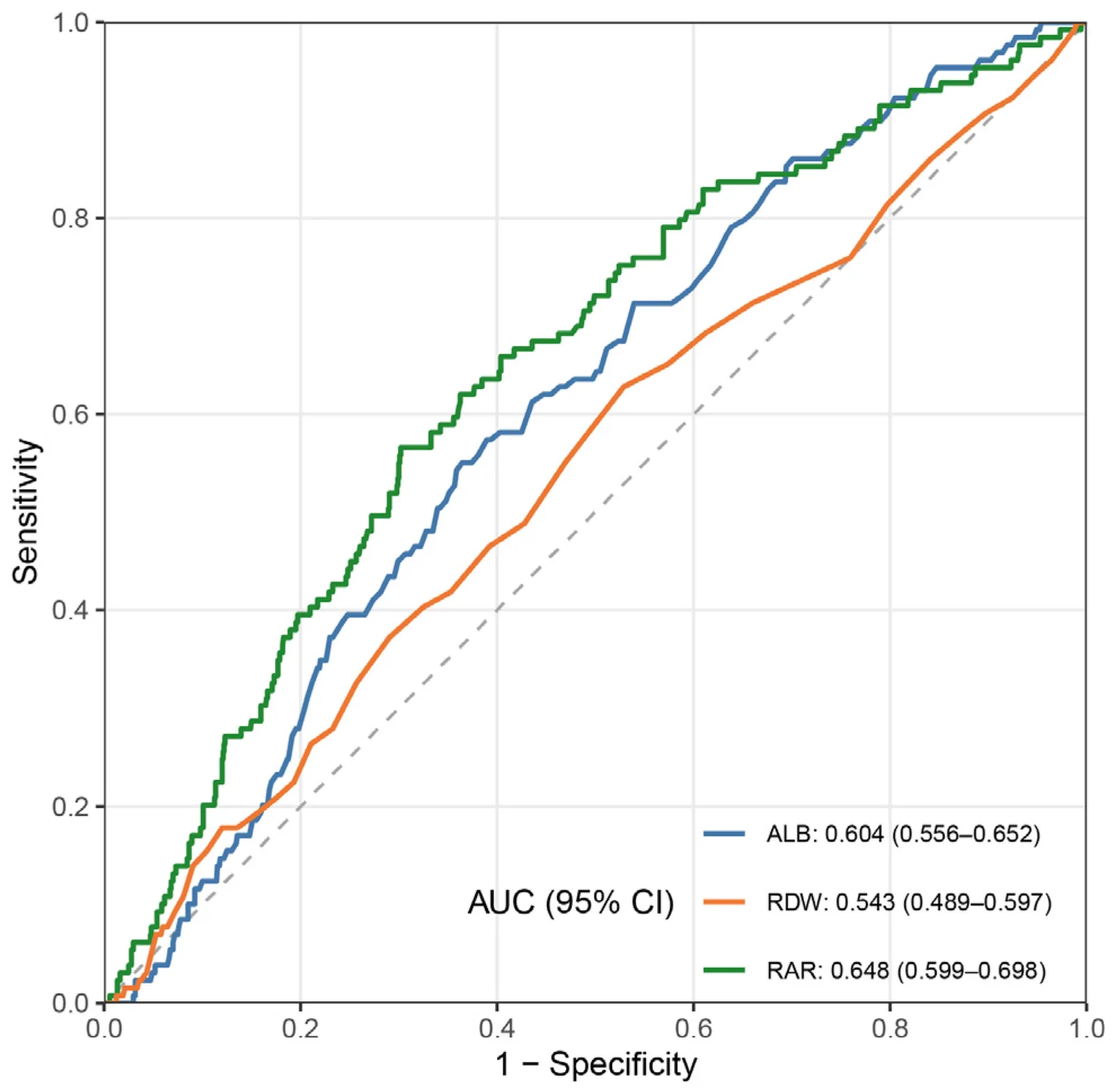
Receiver operating characteristic (ROC) curves of RAR, RDW, and ALB for discriminating the composite renal endpoint in patients with IgAN. Abbreviations: RAR, red blood cell distribution width-to-albumin ratio; RDW, red blood cell distribution width; ALB, serum albumin; AUC, area under the curve; ROC, receiver operating characteristic; IgAN, immunoglobulin A nephropathy.

A total of 1590 patients with biopsy-proven IgAN were screened. After excluding 4 patients with ESRD, 88 with incomplete pathological data, 45 with secondary IgAN, malignant tumors, or systemic diseases, 45 with a follow-up duration of less than 12 months, and 239 with incomplete baseline data, 1169 patients were included in this study (Figure 1). ROC analysis showed that RAR had a higher AUC than albumin and RDW [0.648 (95% CI 0.599–0.698) vs. 0.604 (95% CI 0.556–0.652) and 0.543 (95% CI 0.489–0.597), respectively]. DeLong tests confirmed that the AUC of RAR was significantly higher than that of albumin (*p* = 0.009) and RDW (*p* < 0.001) (Figure 2). Based on the ROC curve, the optimal cutoff of RAR was 0.359, and 782 patients were classified into the low-RAR group and 387 into the high-RAR group (Table 1).

Before PSM, compared with patients in the low-RAR group, those in the high-RAR group were older [36.0 (26.0–44.0) vs. 32.0 (26.0–40.0) years, *p* = 0.002], were less frequently male (32.0% vs. 51.0%, *p* < 0.001), and had higher SBP and a greater prevalence of hypertension. The distribution of CKD stages also differed significantly between groups (*p* < 0.001), with a greater proportion of patients with advanced CKD in the high-RAR group. E1, T1/T2, and C1/C2 lesions were more frequent in the high-RAR group, whereas no significant between-group differences were observed for M1 or S1 lesions. In addition, patients with high RAR had lower hemoglobin, lower eGFR, lower serum albumin, and higher RDW, together with higher TG, TC, 24 h proteinuria, uRBC, HDL-C, and LDL-C levels (all *p* < 0.001). Treatment patterns were also significantly different before matching, with immunosuppressive therapy and/or corticosteroid treatment being more frequent in the high-RAR group (77.5% vs. 51.5%, *p* < 0.001).

After 1:1 PSM, there were 145 patients in each group. Most demographic, clinical, pathological, and treatment characteristics were well balanced between the two groups.

### 3.2. RAR and Renal Composite Outcome

**Figure 3:**
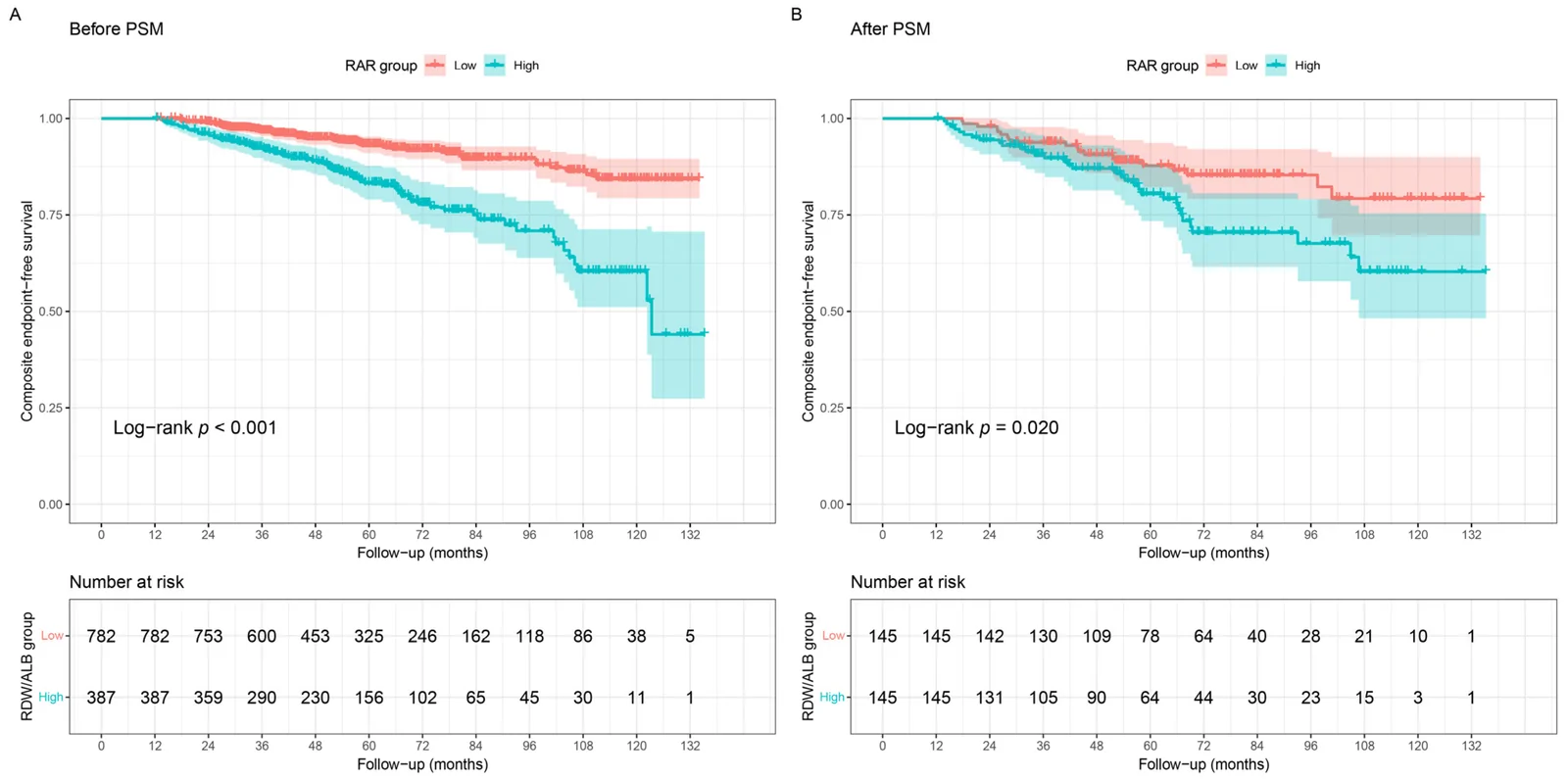
Kaplan–Meier curves for renal outcome between the high-RAR group and the low-RAR group in the unmatched (**A**) and matched (**B**) cohorts. Abbreviations: RAR, red blood cell distribution width-to-albumin ratio; PSM, propensity score matching.

During follow-up, the cumulative incidence of the composite renal endpoint was significantly higher in patients with high RAR than in those with low RAR. Kaplan–Meier analysis showed significantly poorer composite endpoint survival in the high-RAR group before PSM (log-rank *p* < 0.001; Figure 3A). This difference remained significant after PSM, with patients in the high-RAR group continuing to have a lower probability of composite endpoint survival than those in the low-RAR group (log-rank *p* = 0.020; Figure 3B).

Consistent with the Kaplan–Meier analysis, Cox regression showed that high RAR was significantly associated with the composite renal endpoint (Table 2). Before PSM, no violation of the proportional hazards assumption was detected for RAR or for the global models (all *p* > 0.05). Before PSM, patients with high RAR had a higher risk of reaching the composite endpoint in the unadjusted analysis (HR 2.848, 95% CI 2.010–4.038; *p* < 0.001), and the association remained significant after adjustment for demographic characteristics, clinical parameters, Oxford MEST-C lesions, and treatment (Model 4: HR 1.880, 95% CI 1.222–2.893; *p* = 0.004). Similar findings were observed when RAR was analyzed as a continuous variable. In Model 4, RAR was independently associated with the adverse composite renal endpoint (HR 1.371, 95% CI 1.099–1.711; *p* = 0.005).

In the matched cohort, high RAR remained associated with the composite renal endpoint in the unadjusted model (HR 1.917, 95% CI 1.099–3.342; *p* = 0.020). The proportional hazards assumption was assessed using scaled Schoenfeld residuals. No violation was detected for RAR in any model. However, the global tests indicated violations in Models 3 and 4 after PSM (*p* = 0.030 and *p* = 0.015, respectively). Time-varying effects were therefore incorporated for the covariates contributing to the violations [34,35]. After accounting for these effects, high RAR remained significantly associated with the composite renal endpoint in Model 4 (HR 2.128, 95% CI 1.067–4.243; *p* = 0.032). Similarly, when analyzed continuously, RAR was independently associated with adverse renal outcomes (HR 1.989, 95% CI 1.199–3.299; *p* = 0.008).

**Figure 4:**
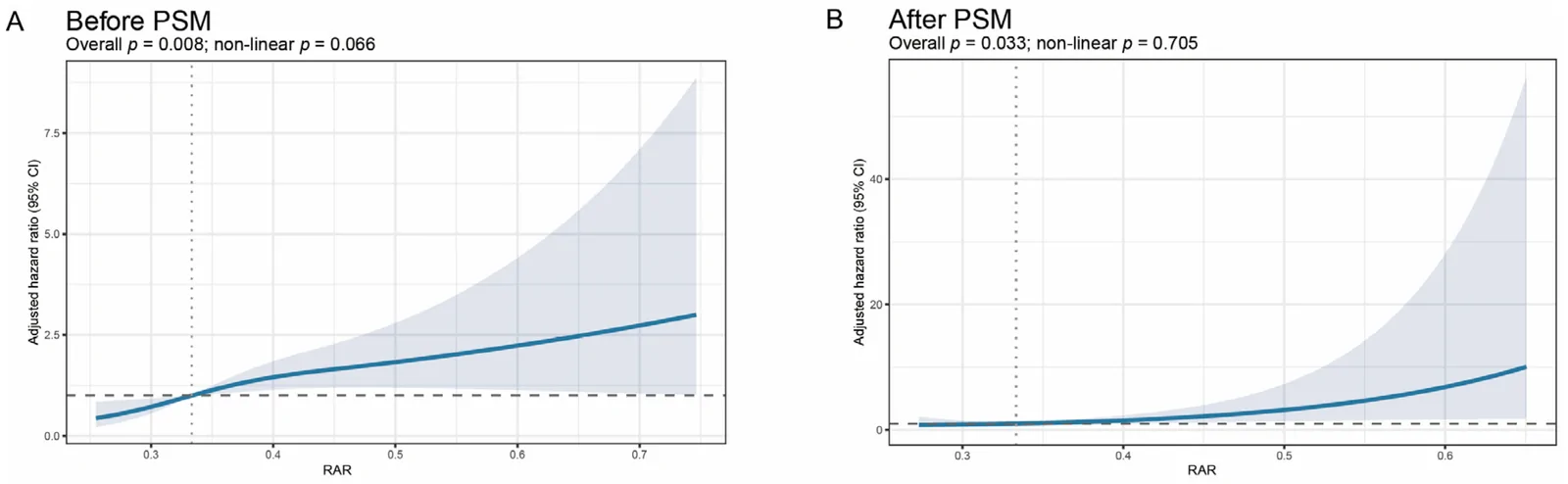
Restricted cubic spline analysis of the association between RAR and the composite renal endpoint before (**A**) and after (**B**) PSM in the fully adjusted model. Abbreviations: RAR, red blood cell distribution width-to-albumin ratio; CI, confidence interval; PSM, propensity score matching.

RCS analysis further showed significant overall associations between RAR and the composite renal endpoint both before PSM (overall *p* = 0.008) and after PSM (overall *p* = 0.033), with no statistically significant evidence of nonlinearity (*p* for nonlinearity = 0.066 and 0.705, respectively) (Figure 4).

### 3.3. Subgroup Analyses

**Figure 5:**
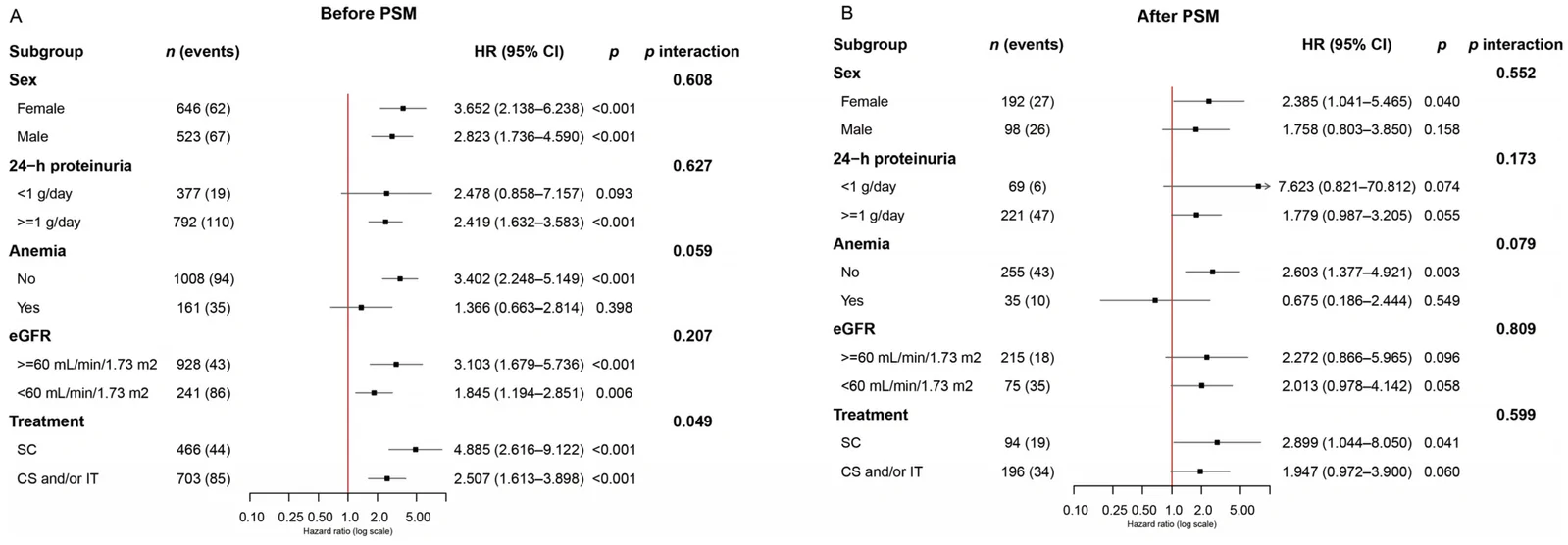
Subgroup analyses of the association between RAR and the composite renal endpoint before (**A**) and after (**B**) propensity score matching (PSM). Note: Hazard ratio (HR) and 95% confidence interval (CI) were analyzed by a Cox regression model. Abbreviations: RAR, red blood cell distribution width-to-albumin ratio; PSM, propensity score matching; HR, hazard ratio; CI, confidence interval.

Subgroup analyses showed that the association between RAR and the composite renal outcome was generally similar across the prespecified subgroups (Figure 5). Before PSM, there were no significant interactions for sex, proteinuria, anemia, or eGFR. A significant interaction was found for treatment strategy (*p* for interaction = 0.049). After PSM, no significant interactions were found in any subgroup, and the treatment interaction seen before matching was no longer present; therefore, the treatment interaction should be interpreted cautiously. Overall, the subgroup results did not show clear differences in the association between RAR and renal outcomes across different patient groups.

## 4. Discussion

In this study, higher RAR was consistently associated with adverse renal outcomes in patients with IgAN. This association remained significant after adjustment for demographic, clinical, pathological, and treatment-related factors and was further supported by PSM analysis. Patients with higher RAR also showed poorer renal survival in Kaplan–Meier analysis before and after matching. In addition, subgroup analyses showed a generally consistent direction of association across different clinical subgroups. These findings support an independent association between higher RAR and adverse renal outcomes in IgAN.

In recent years, increasing attention has been paid to biomarkers that may help characterize disease severity and renal prognosis in IgAN [7,8,9,10,36]. In addition to traditional clinical and pathological factors, several laboratory-based indices have been investigated because they may reflect different biological processes involved in disease progression. Our previous studies of IgAN patients from West China Hospital have evaluated markers related to systemic inflammation, metabolic status, platelet-related changes, and complement activation. Higher neutrophil-to-lymphocyte ratio and systemic inflammatory response index were associated with poorer renal outcomes, while the triglyceride–glucose index suggested a possible role of metabolic abnormalities [7,9,10]. The platelet-to-albumin ratio was also associated with adverse renal outcomes, and mesangial C3 and C1q deposition provided additional pathological information related to complement activation [8,36]. These findings support the value of exploring simple biomarkers from different biological domains in IgAN. The present study extends this line of research by focusing on RAR, which incorporates hematological changes, inflammation or oxidative stress, and nutritional status.

The molecular mechanism between RAR and IgAN has not been identified yet. IgAN is an immune-mediated glomerular disease, and persistent mesangial injury, inflammation, oxidative stress, and tubulointerstitial damage are important in disease progression [3,37]. Increased oxidative stress has been reported in IgAN and may contribute to glomerular and tubular injury [38,39]. RDW may partly reflect these systemic changes, because inflammation and oxidative stress can affect red blood cell production, iron metabolism, and red blood cell stability, leading to greater variation in red blood cell size [40]. Serum albumin may provide additional information on disease severity in IgAN. Lower albumin levels are closely related to proteinuria and may reflect both urinary protein loss and ongoing inflammatory activity. Previous studies in IgAN have shown that low serum albumin is associated with poorer renal survival and may remain prognostically important even after accounting for other clinical factors [41]. Persistent hematuria has also been associated with lower serum albumin, more severe renal pathological lesions, and worse renal outcomes, particularly when accompanied by proteinuria and hypoalbuminemia [29]. These findings suggest that reduced albumin in IgAN may reflect not only nutritional status but also persistent glomerular injury and disease activity. By combining RDW and albumin, RAR may therefore reflect both systemic inflammation and oxidative stress and the severity of renal injury. This concept is supported by previous studies in kidney disease. In a cohort of 997 patients with CKD, higher RAR was associated with a greater risk of ESRD, and patients in the highest tertile had a nearly threefold higher risk than those in the lowest tertile [14]. Although those findings were obtained in a broader CKD population, they support an association between RAR and renal prognosis. Similar associations have also been reported in patients undergoing peritoneal dialysis, in whom higher RAR was related to greater risks of treatment failure and all-cause mortality [42]. These findings suggest that RAR may provide useful prognostic information across different stages of kidney disease. However, most previous studies were conducted in mixed CKD or dialysis populations, in which the underlying causes of kidney disease were heterogeneous. Evidence on IgAN remains limited. In our study, the association between higher RAR and poorer renal outcomes remained significant after adjustment for proteinuria, renal function, hematuria, Oxford MEST-C lesions, and treatment.

Beyond kidney disease, RAR has also been associated with poorer outcomes in several other diseases. In a large population-based cohort, higher RAR was independently associated with increased all-cause mortality and mortality from cardiovascular, cerebrovascular, respiratory, metabolic, and malignant diseases [43]. Similar findings have been reported in cardiovascular disease and Parkinson’s disease [11,44]. Together, these findings suggest that RAR may reflect a broad combination of systemic inflammation, nutritional status, and overall disease burden, which may also explain its prognostic value in IgAN.

This study also had several limitations. First, this was a single-center retrospective analysis with a relatively limited follow-up period. Second, the RAR cutoff was derived from the present cohort and may be subject to optimism. Although consistent associations were observed when RAR was analyzed continuously, the cutoff requires external validation before clinical application. Third, residual confounding cannot be completely excluded despite multivariable adjustment and PSM. Fourth, the limited number of renal events after PSM may have increased the risk of overfitting in the fully adjusted Cox models. Fifth, follow-up duration was not included in the PSM because it was not a baseline characteristic; therefore, the difference in follow-up duration between the matched groups may have influenced the time-to-event analyses. Sixth, although RAR showed better discrimination than RDW or albumin alone, its overall discriminatory performance remained modest. Finally, the subgroup analyses were exploratory and should be interpreted cautiously because of multiple comparisons and the limited number of events in some subgroups. Larger multicenter prospective studies with longer follow-up are needed to confirm these findings and further evaluate the clinical value of RAR in IgAN.

## 5. Conclusions

Higher RAR was independently associated with adverse renal outcomes in patients with IgAN. Further external validation and large multicenter prospective studies are needed to determine its clinical value.

## Figures and Tables

**Table 1:** Baseline characteristics of enrolled IgAN patients.

Variable	Before PSM				After PSM			
	Low RAR	High RAR	*p* Value	SMD	Low RAR	High RAR	*p* Value	SMD
	(*n* = 782)	(*n* = 387)			(*n* = 145)	(*n* = 145)		
Age (years)	32.0 (26.0, 40.0)	36.0 (26.0, 44.0)	0.002	0.235	34.0 (26.0, 41.0)	36.0 (27.0, 43.0)	0.205	0.098
Sex (male), *n* (%)	399 (51.0)	124 (32.0)	<0.001	0.393	51 (35.2)	47 (32.4)	0.619	0.057
SBP (mmHg)	124.0 (114.0, 136.0)	127.0 (115.0, 139.5)	0.041	0.162	125.0 (117.0, 139.0)	126.0 (115.0, 140.0)	0.804	0.032
DBP (mmHg)	81.0 (74.2, 90.0)	82.0 (75.0, 92.0)	0.228	0.095	80.0 (74.0, 90.0)	80.0 (72.0, 92.0)	0.998	0.033
Hypertension, *n* (%)	199 (25.4)	120 (31.0)	0.045	0.124	42 (29.0)	49 (33.8)	0.376	0.107
CKD stage, *n* (%)			<0.001				0.966	
Stage 1	448 (57.3)	175 (45.2)		0.243	68 (46.9)	68 (46.9)		<0.001
Stage 2	209 (26.7)	96 (24.8)		0.044	41 (28.3)	38 (26.2)		0.047
Stage 3	114 (14.6)	93 (24.0)		0.241	31 (21.4)	33 (22.8)		0.035
Stage 4	11 (1.4)	23 (5.9)		0.243	5 (3.4)	6 (4.1)		0.037
Oxford classification								
M1, *n* (%)	604 (77.2)	318 (82.2)	0.052	0.123	121 (83.4)	118 (81.4)	0.644	0.052
E1, *n* (%)	15 (1.9)	38 (9.8)	<0.001	0.341	5 (3.4)	8 (5.5)	0.395	0.089
S1, *n* (%)	470 (60.1)	239 (61.8)	0.586	0.034	91 (62.8)	90 (62.1)	0.904	0.014
T1/T2, *n* (%)	133 (17.0)	108 (27.9)	<0.001	0.263	36 (24.8)	35 (24.1)	0.891	0.017
C1/C2, *n* (%)	149 (19.1)	107 (27.6)	<0.001	0.204	39 (26.9)	35 (24.1)	0.59	0.066
Clinical parameters								
Hb (g/L)	137.0 ± 17.6	123.6 ± 21.6	<0.001	0.679	129.0 (118.0, 138.0)	127.0 (117.0, 140.0)	0.66	0.012
Cr (µmol/L)	83.0 (65.0, 104.0)	85.6 (64.0, 123.5)	0.21	0.226	83.7 (66.0, 116.8)	84.7 (63.0, 115.1)	0.597	0.011
eGFR (mL/min/1.73 m^2^)	96.2 (73.0, 118.2)	81.7 (52.7, 116.5)	<0.001	0.297	88.2 (60.9, 114.2)	83.0 (56.0, 118.0)	0.995	0.010
ALB (g/L)	42.1 (39.6, 44.5)	33.9 (29.3, 36.8)	<0.001	1.921	40.7 (38.7, 43.1)	35.0 (32.6, 37.6)	<0.001	1.225
RDW (%)	13.0 (12.5, 13.5)	13.9 (13.2, 14.7)	<0.001	0.899	13.0 (12.6, 13.5)	14.0 (13.3, 14.9)	<0.001	0.963
TG (mmol/L)	1.4 (1.0, 2.1)	1.6 (1.1, 2.4)	<0.001	0.177	1.5 (1.0, 2.2)	1.5 (1.0, 2.1)	0.95	0.082
TC (mmol/L)	4.6 (4.0, 5.3)	5.3 (4.4, 6.6)	<0.001	0.638	4.9 (4.2, 6.0)	4.9 (4.0, 5.7)	0.306	0.061
24 h proteinuria (g/day)	1.02 (0.61, 2.00)	2.68 (1.50, 4.54)	<0.001	0.798	1.80 (1.00, 3.00)	1.93 (1.00, 2.65)	0.891	0.003
uRBC (/HPF)	17.0 (6.0, 53.0)	25.0 (7.5, 85.5)	<0.001	0.228	26.0 (9.0, 74.0)	16.0 (6.0, 59.0)	0.102	0.012
UA (µmol/L)	369.5 (303.1, 447.6)	355.1 (296.5, 434.0)	0.077	0.094	356.0 (295.0, 430.9)	354.0 (291.0, 427.0)	0.806	0.041
HDL-C (mmol/L)	1.33 (1.08, 1.62)	1.43 (1.15, 1.88)	<0.001	0.367	1.43 (1.18, 1.69)	1.36 (1.13, 1.77)	0.327	0.040
LDL-C (mmol/L)	2.58 (2.07, 3.18)	3.10 (2.39, 4.05)	<0.001	0.563	2.90 (2.35, 3.66)	2.73 (2.23, 3.45)	0.158	0.101
Treatment, *n* (%)			<0.001				0.802	
Support treatment	379 (48.5)	87 (22.5)		0.564	46 (31.7)	48 (33.1)		0.030
Immunosuppression and/or steroids	403 (51.5)	300 (77.5)		0.564	99 (68.3)	97 (66.9)		0.030
Follow-up and outcomes								
Composite endpoint, *n* (%)	56 (7.2)	73 (18.9)	<0.001	0.353	20 (13.8)	33 (22.8)	0.048	0.271
Observed follow-up (months)	54.8 (36.7, 79.0)	54.0 (36.1, 72.8)	0.26	0.096	65.5 (49.3, 85.7)	56.9 (34.7, 78.6)	0.009	0.317

*p* values were calculated using Student’s *t* test for normally distributed continuous variables, the Mann–Whitney U test for non-normally distributed continuous variables, and the χ^2^ test or Fisher’s exact test for categorical variables. Standardized mean differences were used to assess covariate balance before and after PSM, with an absolute SMD < 0.20 considered as adequate balance. Abbreviations: RAR, red blood cell distribution width-to-albumin ratio; PSM, propensity score matching; SMD, standardized mean difference; SBP, systolic blood pressure; DBP, diastolic blood pressure; CKD, chronic kidney disease; M, mesangial proliferation; E, endocapillary proliferation; S, segmental glomerulosclerosis; T, tubular atrophy or interstitial fibrosis; C, crescents; Hb, hemoglobin; Cr, serum creatinine; eGFR, estimated glomerular filtration rate; ALB, albumin; RDW, red blood cell distribution width; TG, total triglyceride; TC, total cholesterol; uRBC, urinary red blood cell counts; UA, uric acid; LDL-C, low-density lipoprotein cholesterol; HDL-C, high-density lipoprotein cholesterol.

**Table 2:** Unadjusted and adjusted Cox regression model for the renal outcome in patients with IgAN in the unmatched and matched cohorts.

	Before PSM				After PSM			
	Higher RAR		Continuous Variable		Higher RAR		Continuous Variable	
	HR (95% CI)	*p*	HR (95% CI)	*p*	HR (95% CI)	*p*	HR (95% CI)	*p*
Unadjusted	2.848	<0.001	1.238	<0.001	1.917	0.02	1.298	0.043
	(2.010–4.038)		(1.108–1.383)		(1.099–3.342)		(1.008–1.671)	
Model 1	3.177	<0.001	1.271	<0.001	2.072	0.01	1.341	0.024
	(2.224–4.538)		(1.136–1.421)		(1.181–3.634)		(1.040–1.729)	
Model 2	2.219	<0.001	1.375	0.004	2.22	0.015	2.22	0.001
	(1.448–3.400)		(1.108–1.706)		(1.169–4.217)		(1.376–3.582)	
Model 3	1.969	0.002	1.377	0.004	2.441	0.009	2.203	0.002
	(1.285–3.018)		(1.106–1.716)		(1.250–4.769)		(1.346–3.604)	
Model 4	1.88	0.004	1.371	0.005	2.128	0.032	1.989	0.008
	(1.222–2.893)		(1.099–1.711)		(1.067–4.243)		(1.199–3.299)	

RAR was analyzed both as a categorical variable (lower RAR vs. higher RAR) and continuous variable. Data are reported as hazard ratio (HR) and 95% confidence interval (CI). Model 1 was, adjusted for baseline age and gender; Model 2 was, adjusted for covariates in Model 1 plus clinical parameters (24 h-proteinuria, eGFR < 60 mL/min/1.73 m^2^, hematuria, SBP, DBP, uric acid, HDL-C, LDL-C, anemia, hypoalbuminemia, hypertriglyceridemia, and hypercholesterolemia); Model 3 was, adjusted for covariates in Model 2 plus pathological lesions (MEST-C); and Model 4 was, adjusted for covariates in Model 3 plus treatment. In the matched cohort, time-varying effects for SBP and DBP were incorporated into Models 2, 3 and 4 using interactions with log(time) to account for violations of the proportional hazards assumption. Abbreviations: RAR, red blood cell distribution width-to-albumin ratio; PSM, propensity score matching; HR, hazard ratio; CI, confidence interval.

## Data Availability

Data regarding the study are available upon request to the corresponding author.
